# Medial septum activation produces opposite effects on dopamine neuron activity in the ventral tegmental area and substantia nigra in MAM vs. normal rats

**DOI:** 10.1038/s41537-018-0059-3

**Published:** 2018-09-03

**Authors:** David M. Bortz, Anthony A. Grace

**Affiliations:** 1 Department of Neuroscience, University of Pittsburgh, Pittsburgh, PA 15260 USA; 2 Department of Psychiatry, University of Pittsburgh, Pittsburgh, PA 15260 USA; 3 Department of Psychology, University of Pittsburgh, Pittsburgh, PA 15260 USA

## Abstract

The medial septum (MS) differentially impacts midbrain dopamine (DA) neuron activity via the ventral hippocampus, a region implicated in DA-related disorders. However, whether MS regulation of ventral tegmental area (VTA) and substantia nigra pars compacta (SNc) is disrupted in a developmental disruption model of schizophrenia is unknown. Male Sprague-Dawley rats were exposed at gestational day 17 to methylazoxymethanol (MAM) or saline. As adults, NMDA (0.75 µg/0.2 µL) was infused into the MS, and either DA neuron activity in the VTA and SNc (7–9 anesthetized rats per group) or amphetamine-induced hyperlocomotion (AIH, 11-13 rats per group) was measured. MS activation produced a 58% increase in the number of spontaneously active DA neurons in VTA and a 37% decrease in SNc in saline rats. However, MS activation produced opposite effects on DA population activity in MAM rats, decreasing VTA DA activity by 51% and increasing SNc DA activity by 47%. MS activation also increased AIH by 113% in MAM rats, opposite of what is seen in intact rats. The effect in behavioral output may be due to disrupted GABAergic regulation of SNc as bicuculline infusion into vSub, which selectively prevented the MS activation-induced decrease in SNc DA activity in intact rats, prevented the increase in AIH and SNc DA activity in MAM rats. These findings demonstrate that the regulation of midbrain DA neurons by the MS is disrupted in this well-validated animal model, suggesting that it could be a potential locus for pharmacological intervention in disorders such as schizophrenia.

## Introduction

Schizophrenia is a devastating disorder characterized by dopamine (DA) dysregulation in human patients^1,2^ and rodent models.^3–5^ This dysregulation is thought to be caused by pathology within the upstream circuits that control DA activity, such as the hippocampus^6–9^, rather than in the DA system itself.^5,10–12^ As such, characterizing the circuits that regulate DA transmission in intact animals and animal models remains a high priority.

One brain region that is a potent regulator of DA neuron activity in both the ventral tegmental area (VTA) and substantia nigra pars compacta (SNc) is the medial septum (MS).^13^ The MS is a sub-region of the basal forebrain that regulates DA transmission via GABAergic and cholinergic projections to the hippocampus;^13–15^ two projections that are critical for hippocampal theta activity^16,17^ and spatial learning and memory tasks.^18–20^ We showed previously that MS activation increased the number of spontaneously active DA neurons in the VTA via cholinergic inputs to the ventral subiculum of the hippocampus (vSub), decreased the number of active DA neurons in the SNc via GABAergic inputs to vSub, and inhibited amphetamine-induced hyperlocomotion (AIH).^13^ The behaviorally relevant output of the DA system is proposed to be phasic burst-firing, driven by NMDA stimulation of midbrain DA neurons from the pedunculopontine tegmentum.^21^ However, for a DA neuron to burst fire, the neuron must be depolarized and spontaneously active; if it is hyperpolarized the magnesium block of the NMDA channel prevents NMDA-driven burst firing.^21–23^ Therefore, by regulating the number of neurons that are spontaneously active, the MS to midbrain pathway controls the “gain” of the burst firing “signal”, effectively setting a “context” for behaviorally relevant DA transmission from both the VTA and SNc.^13,24^

The pathway by which the MS regulates DA activity in both the VTA and SNc requires the vSub, a region that has been heavily implicated in schizophrenia.^3,5–7^ Furthermore, the GABAergic projections from MS are known to contact parvalbumin-positive interneurons in the hippocampus,^14,25^ which is the interneuron class that has been specifically implicated in the hyperactive hippocampal activity and downstream-elevated DA release seen in patients^26,27^ and animal models.^28^ Therefore, it is important to further examine the regulation of DA transmission by the MS in an animal model that recapitulates the constructs of decreased parvalbumin interneurons and hippocampal hyperactivity in order to determine if the MS could be a potential locus for disruption in schizophrenia.

Thus, we examined the MS to midbrain pathway in a well-validated animal model of hippocampal-dependent DA disruption—by prenatal (GD17) administration of methylazoxymethanol acetate (MAM).^5,29^ We performed electrophysiological recordings of VTA and SNc spontaneous DA activity in MAM and saline-treated (Sal) rats after NMDA or vehicle infusion into the MS, and examined the behavioral consequence of MS activation using the AIH paradigm.

## Results

### MAM treatment causes MS activation to produce opposite effects on VTA and SNc DA neuron activity compared to Sal rats

**Fig. 1:**
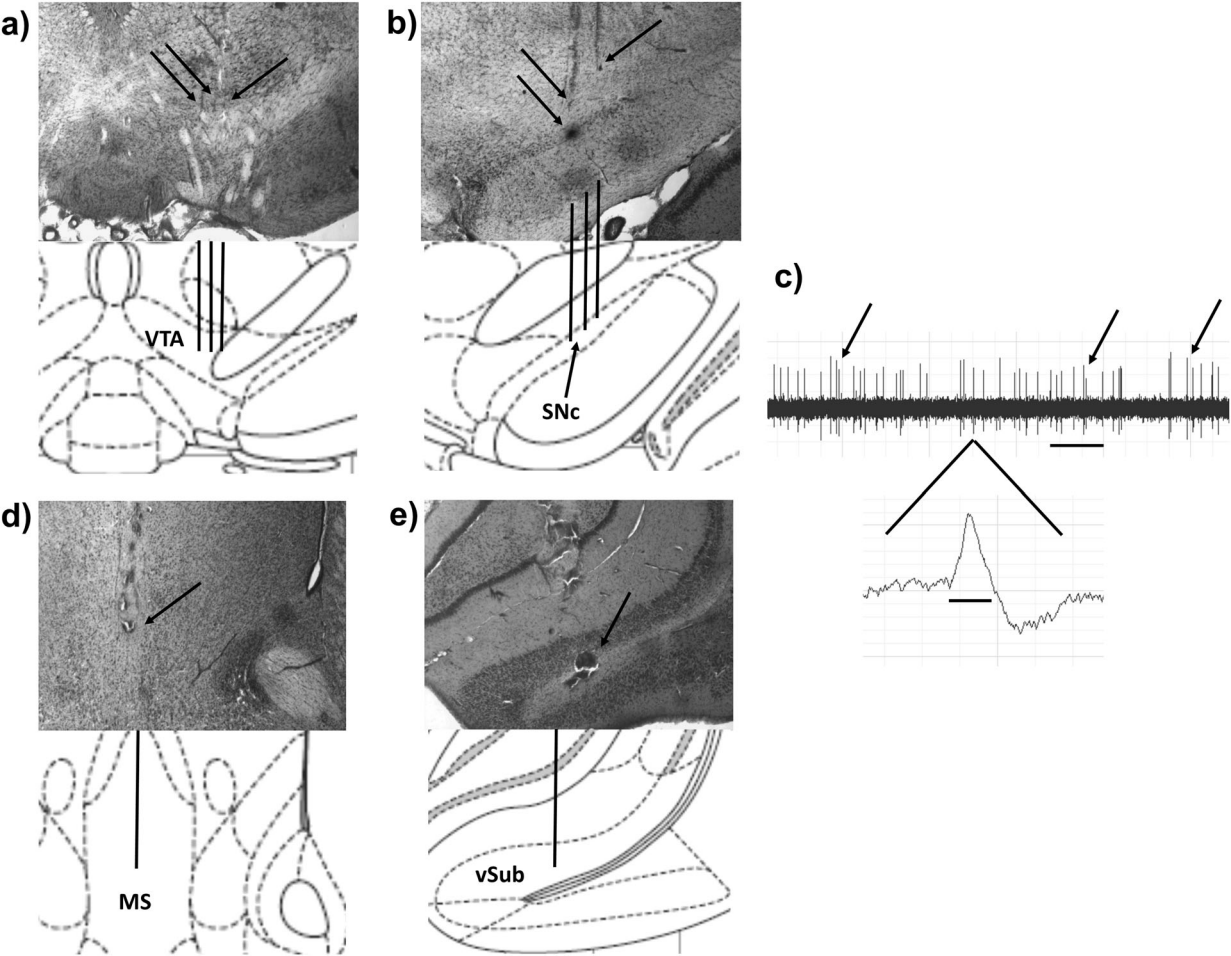
Representative placements, DA neuron identification, and drug infusion locations. **a** Population activity in the VTA was measured in nine sequential electrode tracks between AP −5.3 to 5.7 and ML 0.6–1.0. Arrows indicate three tracks across the medial–lateral span of the VTA. **b** Population activity is measured similarly in the SNc, between AP −4.9 to 5.3 and ML 2.0–2.4. Following the conclusion of the 9th track (top arrows), Chicago Sky Blue is ejected from the electrode leaving a blue dot for histological verification (bottom arrow). **c** Representative DA recording from the VTA and individual spike. The time bar for representative tracing equals 1 s, demonstrating a frequency of approximately six spikes per second. Arrows indicate three sets of spikes that are occurring in bursts. The time bar under the individual spike is 1.1 ms. Representative histology and matching illustration for the target region of the **d** MS and **e** vSub. The line in the illustration denotes the cannula placement and the arrow indicates the ventral termination of the cannula track

**Fig. 2:**
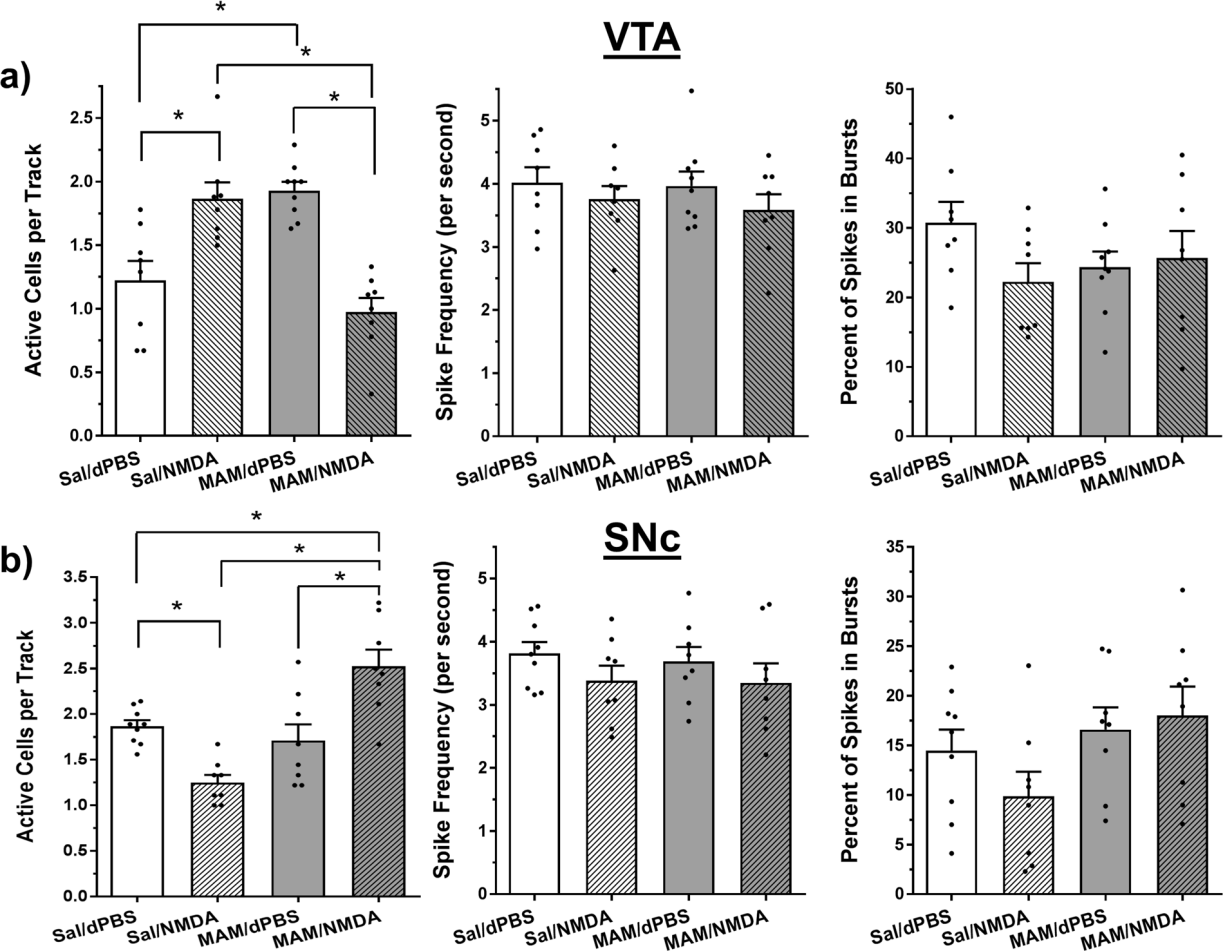
MS activation produced opposite effects on DA neuron activity in saline vs. MAM rats. **a** NMDA activation of the MS increased DA neuron population activity in the VTA compared to vehicle (dPBS; mean ± SEM). MAM rats displayed elevated DA activity compared to Sal animals, but showed an opposite decrease in DA activity following MS activation (*Tukey’s *P* < 0.05). Spike frequency and bursting activity in VTA were not affected by MAM treatment or MS activation. **b** NMDA activation of the MS decreased DA neuron population activity in the SNc compared to vehicle (dPBS). DA population activity in the SNc of MAM rats was not different from Sal rats at baseline, but showed an opposite increase in DA activity following MS activation (*Tukey’s *P* < 0.05). Spike frequency and bursting activity in SNc were not affected by MAM treatment or MS activation. Black dots indicate data from individual rats (*N* = 8–9 rats per group in both VTA and SNc)

To determine if the MS regulation of VTA and SNc DA neuron activity is disrupted in the MAM model, adult male anesthetized MAM and Sal rats (*N* = 8–9 rats per group) were infused with NMDA (0.75 µg in 0.2 µL) or dPBS into the MS (Fig. 1d) and DA neurons were recorded in the VTA or SNc in nine sequential tracks (Fig. 1a,b). MS activation produced significantly different effects on DA neuron activity in MAM vs. Sal rats in the VTA (overall ANOVA: *F*_3, 29_ = 16.12, *P* < 0.0001, see Fig. 2a). In Sal rats, MS activation produced a 58% increase in the number of active DA neurons per recording track in the VTA (mean ± SEM; Sal/dPBS: 1.2 ± 0.2, Sal/NMDA: 1.9 ± 0.1 neurons/track, Tukey’s: *P* = 0.0037), similar to what has been shown previously in intact animals.^13^ In contrast, MAM rats, whose elevated basal level of DA neuron activity (MAM/dPBS: 1.9 ± 0.1 neurons/track) has been shown in previous studies,^3^ displayed an opposite 47% reduction in the number of active DA neurons recorded in the VTA after MS activation (MAM/NMDA: 1.0 ± 0.1 neurons/track, Tukey’s: *P* < 0.0001; Tukey’s Sal/NMDA vs. MAM/NMDA: *P* < 0.0001; Table 1). Spike frequency and the percentage of spikes in bursts in the VTA were not affected by MAM treatment or MS activation (*P* > 0.1, Fig. 2A).

**Table 1:** Summary of changes in DA neuron activity and AIH in different treatment groups

Treatment	MS infusion	vSub infusion	VTA DA activity	SNc DA activity	AIH
Intact^13^	dPBS	N/A	1.0 ± 0.1	1.7 ± 0.1	N/A
Intact^13^	NMDA	N/A	1.8 ± 0.1;↑	1.1±0.2;↓	↓
Sal	dPBS	N/A	1.2±0.2; –	1.9 ± 0.1; –	–
Sal	NMDA	N/A	1.9 ± 0.1;↑	1.2±0.1↓	↓
MAM	dPBS	N/A	1.9 ± 0.1;↑	1.7 ± 0.2; –	–
MAM	NMDA	N/A	1.0 ± 0.1; –	2.5 ± 0.2;↑	↑
MAM	NMDA	Bicuculline	N/A	1.4 ± 0.2; –	–
MAM	dPBS	Bicuculline	N/A	1.7 ± 0.2; –	–

Numbers indicate the neurons/track mean ± SEM for that treatment group. Symbols denote the change (increase, decrease, or no change) in the respective category *compared to the intact/dPBS group* published previously^13^

MS activation also produced significantly different effects on DA neuron activity in MAM vs. Sal rats in the SNc (overall ANOVA: *F*_3, 29_ = 14.82, *P* < 0.0001, Fig. 2B). MS activation produced a 37% decrease in the number of active DA neurons per recording track in Sal rats (Sal/dPBS: 1.9 ± 0.1, Sal/NMDA: 1.2 ± 0.1 neurons/track, Tukey’s: *P* = 0.0136), similar to what has been shown previously in intact animals.^13^ Different from what has been reported in the VTA, MAM rats showed no effect of MAM treatment on basal spontaneous DA neuron activity in the SNc (MAM/dPBS: 1.7 ± 0.2 neurons/track). However, as with the VTA, MS activation produced an opposite response in the SNc of MAM rats (47% increase, MAM/NMDA: 2.5 ± 0.2 neurons/track, Tukey’s: *P* = 0.0012) that was significantly different from what was seen in the Sal rats (Tukey’s Sal/NMDA vs. MAM/NMDA: *P* < 0.0001; Table 1). Spike frequency and the percentage of spikes in bursts in SNc were not affected by MAM treatment or MS activation (*P* > 0.1, Fig. 2B).

### MAM treatment causes MS activation to produce opposite effects on AIH compared to Sal rats

**Fig. 3:**
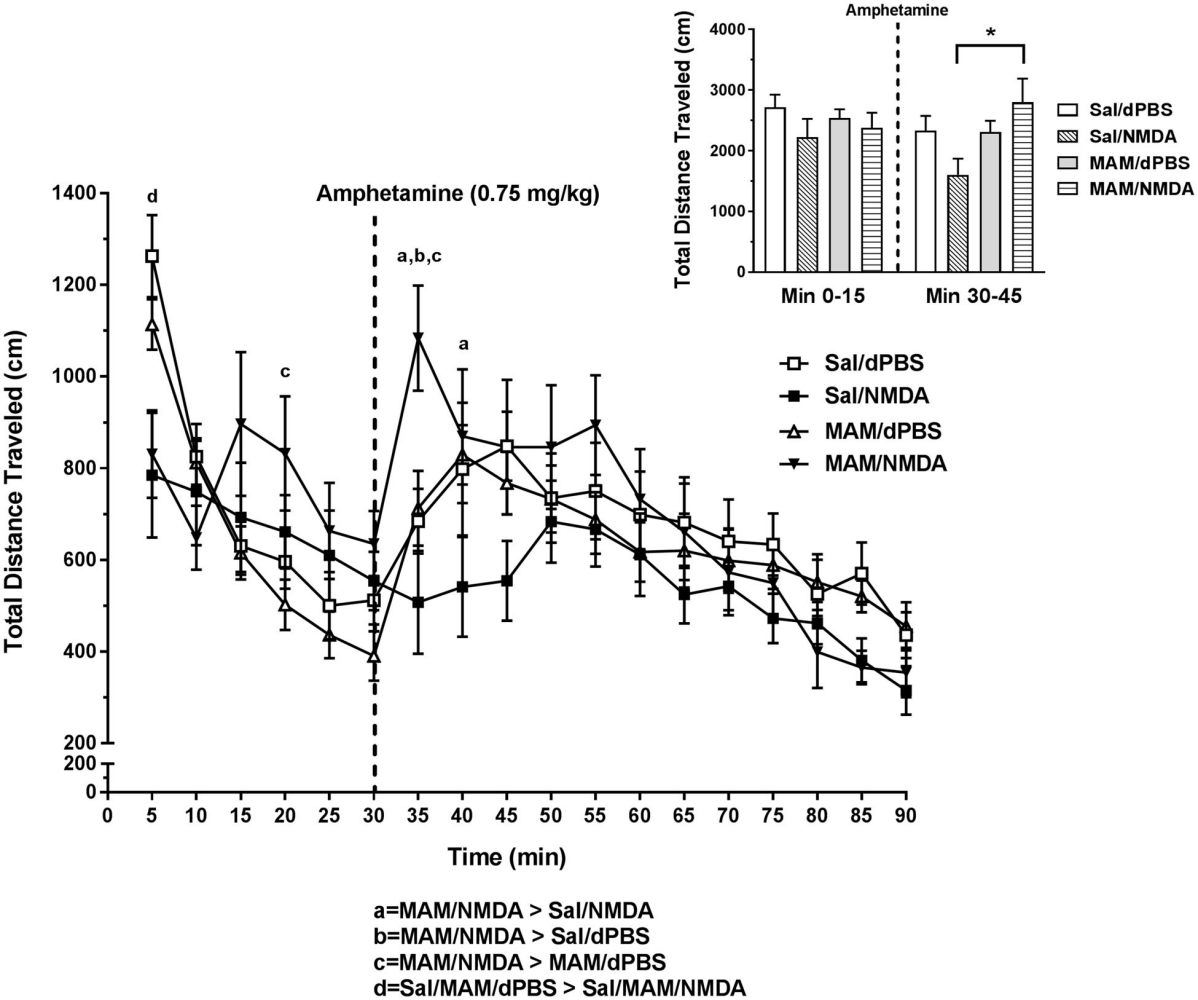
MS activation produced opposite effects on AIH in saline vs. MAM rats. NMDA (0.75 µg in 0.2 µL) activation of the MS significantly reduced basal locomotor behavior at the first time point in both MAM and Sal rats (mean ± SEM; d = Tukey’s *P* < 0.05). MAM rats showed significantly elevated AIH 5 min following amphetamine injection (indicated by dashed line, 0.75 mg/kg; at 35 min), compared to all other groups (a,b,c = Tukey’s *P* < 0.05) and compared to Sal/NMDA rats at minute 40 (a = Tukey’s P < 0.05). Inset: The total distance travelled (cm) for each group is summed over two key intervals—minute 0 to 15 and minute 30 to 45. The dashed line indicates when the amphetamine was infused. MAM/NMDA rats had significantly elevated locomotion in the first 15 min after amphetamine compared to Sal/NMDA rats (*P = 0.009)

To determine the impact of MS activation on locomotor behavior in MAM vs. Sal rats, the rats were infused with NMDA (0.75 µg in 0.2 µL) or dPBS into the MS and the total distance traveled (cm) in an open field arena was measured before and after systemic amphetamine injection (0.75 mg/kg, *N* = 11–13 rats per group). MS activation significantly reduced locomotor behavior before amphetamine injection (at 5 min) compared to Sal/dPBS and MAM/dPBS groups (Tukey’s: *P* < 0.05, see Fig. 3). Following amphetamine injection, MS activation produced opposite effects on locomotor behavior in MAM vs. Sal rats (overall two-way ANOVA (time point × intracranial drug condition): *F*_51, 731_ = 2.429, *P* < 0.0001, Tukey’s Sal/NMDA vs. MAM/NMDA: 35 min: *P* < 0.0001, 40 min: *P* = 0.0435). This effect was primarily caused by a sharp spike in locomotion in the MAM/NMDA rats in the first epoch following amphetamine injection, which was different from all other groups (Tukey’s: *P*’s < 0.05, see Fig. 3) and opposite of the reduction seen following MS activation in Sal and intact rats (Table 1).^13^ The difference in locomotor behavior between MAM and Sal rats following MS activation was not likely driven by changes in stereotypy, as MS activation did induce an increase in stereotypy (main effect of MS activation, *F*_3, 25_ = 4.967, *P* = 0.0077), but it was not different between MAM and Sal rats (*P* > 0.05; see Supplementary Figure 1).

### Effects on AIH and SNc DA activity in MAM rats are mediated by GABAergic inputs to vSub

**Fig. 4:**
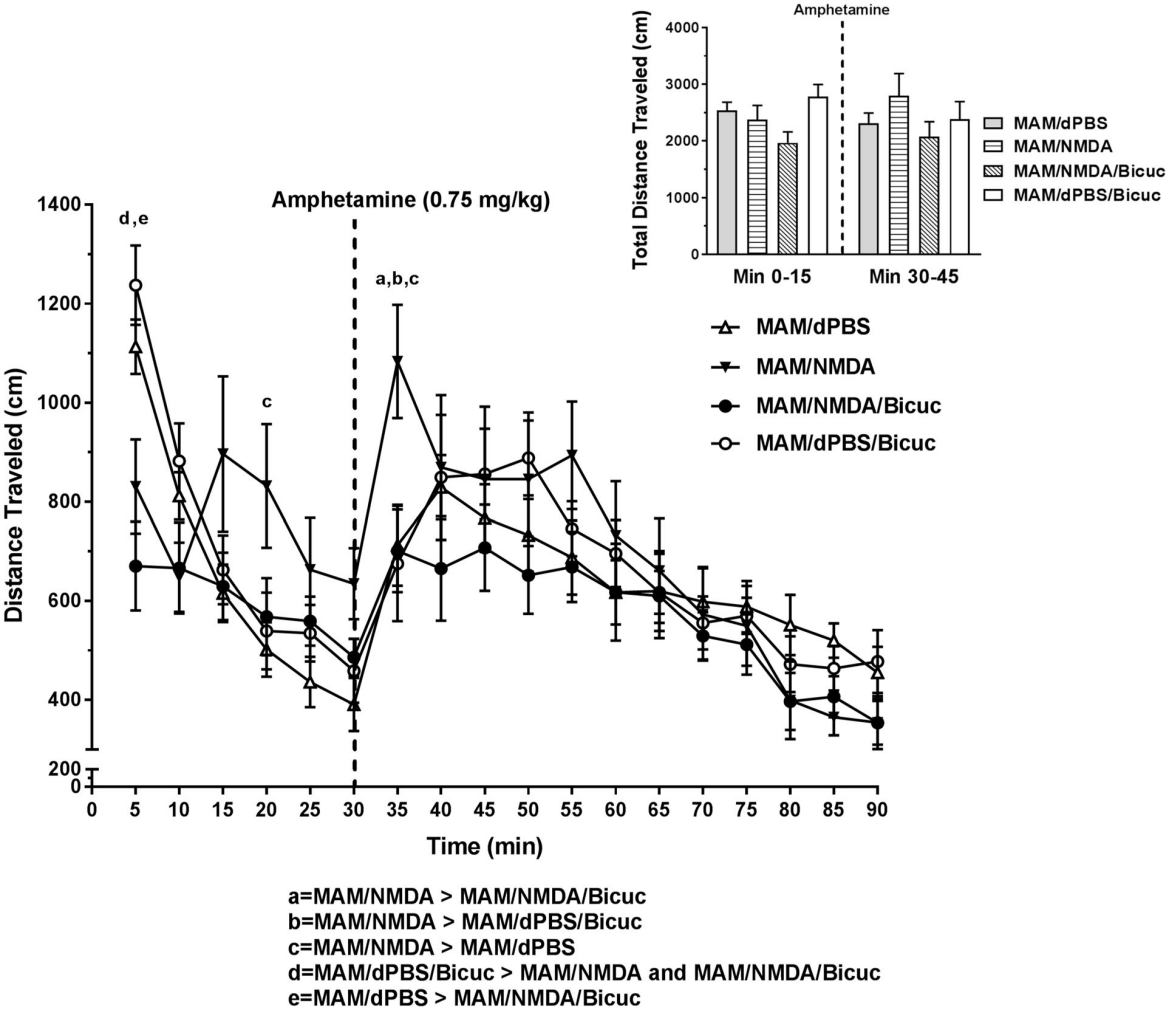
Activation of AIH in MAM rats is blocked by vSub bicuculline infusions. Bicuculline (Bicuc; 12.5 ng in 0.5 µL) infusion into the vSub just prior to MS activation prevented the increase in amphetamine-induced hyperlocomotion (amphetamine injection marked by dashed line), restoring locomotor behavior back to MAM/ dPBS levels (mean ± SEM; a,b,c,d,e = Tukey’s test *P* < 0.05). Inset: The total distance travelled (cm) for each group is summed over two key intervals—minute 0 to 15 and minute 30 to 45. The dashed line indicates when the amphetamine was infused

Our previous studies indicated that vSub bicuculline infusions selectively prevented the MS activation-induced decreases in SNc DA activity and reversed the reduction in AIH in intact rats.^13^ This suggested that these effects were both driven by GABAergic inputs to vSub, likely from the MS. To determine if the disruptions in AIH and SNc DA neuron activity were also mediated by GABAergic inputs to vSub, MAM rats were infused with bicuculline in the vSub (Fig. 1E; *N* = 11–12 rats per group, Bicuc; 12.5 ng in 0.5 µL) just prior to NMDA or dPBS activation of the MS. In accordance with this hypothesis, bicuculline infusion in MAM rats prior to MS activation completely prevented the increase in AIH (Fig. 4, overall two-way ANOVA (time point × intracranial drug condition): *F*_51, 756_ = 1.629, *P* = 0.0044, Tukey’s MAM/NMDA vs. MAM/NMDA/Bicuc: 35 min: *P* < 0.0041; Table 1). Interestingly, the reduction in locomotor behavior in the NMDA-treated rats in the first 5-min epoch was not prevented by bicuculline (Tukey’s vs. MAM/dPBS and MAM/dPBS/Bicuc: *P*’s < 0.05), suggesting this effect is likely mediated via a different pathway.

**Fig. 5:**
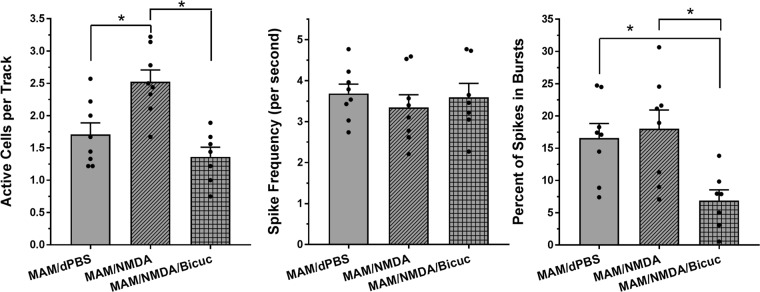
The MS activation-induced increase in SNc DA neuron activity was prevented by vSub bicuculline infusion. Bicuculline (Bicuc; 12.5 ng in 0.5 µL) infusion into the vSub just prior to MS activation prevented the increase in SNc DA neuron activity, with DA activity remaining at MAM/dPBS levels (mean ± SEM; *Tukey’s *P* < 0.05). Bicuculline infusions also significantly reduced the percentage of spikes occurring in bursts compared to both other groups (*Tukey’s *P* < 0.05). Black dots indicate data from individual rats (*N* = 7–8 rats per group)

Bicuculline infusion also prevented the MS activation-induced increase in DA activity in the SNc of MAM rats (*N* = 7 rats; Fig. 5). Bicuculline infusion reduced the number of active DA neurons in the SNc by 44% (MAM/NMDA/Bicuc: 1.4 ± 0.2 neurons/track) to a level comparable to the MAM/dPBS rats (1.7 ± 0.2 neurons/track) and significantly reduced from the MAM/NMDA rats (2.5 ± 0.2 neurons/track, *F*_2, 20_ = 11.8, *P* = 0.0004, Tukey’s: *P* = 0.0004; Table 1). Bicuculline infusion also significantly reduced the percentage of spikes occurring in bursts compared to both other groups (*F*_2, 20_ = 6.184, *P* = 0.0081), but did not affect spike frequency.

## Discussion

These experiments resulted in several findings. First, this study reports that MAM treatment did not impact baseline DA activity in the SNc, which is markedly different than the large increase in baseline activity seen in the VTA in MAM rats. Second, the MS-mediated regulation of VTA and SNc DA neuron activity seen in intact^13^ and Sal-treated rats was disrupted by prenatal MAM treatment. MAM-treated rats displayed a decrease in DA neuron activity in the VTA and an increase in the SNc following MS activation, which is opposite of the pattern seen in intact and Sal rats. Third, MAM rats also displayed the opposite pattern of locomotor activity following amphetamine challenge, with MS activation increasing AIH in MAM rats instead of decreasing it as it did in intact rats. Finally, the increase in AIH was likely driven by a disruption in the GABAergically mediated control of the SNc as bicuculline infusion into the vSub prior to MS activation, which was previously shown to selectively affect SNc DA neuron activity and not VTA DA activity in intact rats,^13^ reversed the increase in both AIH and SNc DA activity in MAM rats.

### Proposed circuit contributing to disruption of MS-DA regulation in MAM rats

The MS has been shown previously to regulate AIH and DA population activity in the VTA and SNc of intact rats via a multi-synaptic pathway (Supplementary Figure 2) that projects through the ventral hippocampus and ventral pallidum.^13^ The ability of this pathway to regulate DA activity has also been shown by direct modulation of the ventral hippocampus,^30^ nucleus accumbens,^30,31^ and ventral pallidum,^13,21,32^ providing evidence for the specific circuit that is likely to mediate the effects shown here. Furthermore, the vSub of the hippocampus has been shown to be critical for the differential regulation of the VTA and SNc by the MS, as direct infusion of cholinergic or GABAergic antagonists into the vSub selectively modulates MS-mediated DA activity in the VTA or SNc, respectively.^13^ Importantly, the manipulations in these papers are not pathway-specific, so other long-loop pathways involving interconnected regions that also affect midbrain DA activity, such as the prefrontal cortex or amygdala,^33–35^ could play a role in the effects reported. However, the ability to modulate DA activity at every brain region in the pathway indicates that the pathway described is the most parsimonious circuit explanation for MS mediation of the VTA and SNc.

Here, we demonstrate that prenatal MAM treatment disrupts MS mediation of both VTA and SNc and AIH. While MAM has been shown to result in limbic and cortical thining,^36^ due primarily to dendritic arbor reduction and loss of restricted classes of interneurons,^28,37^ major brain distortions are not evident. Furthermore, infusion of NMDA to sites directly adjacent to the MS in intact rats did not result in DA activity changes similar to those seen after MS activation.^13^ As such, gross changes to MS connectivity by MAM leading to activation of regions other than the MS is not likely to be the cause of the DA and AIH dysregulation shown here. However, a consistent finding in MAM-treated rats is a significant loss of parvalbumin-positive interneurons in the vSub.^28,37^ This interneuron class has been shown to be one of the cellular targets of GABAergic projections from MS,^14,25^ and the vSub is required for MS-mediated DA activity changes in both VTA and SNc.^13^ Therefore, one possibility is that the differential effect of MS activation on DA activity and AIH in MAM vs. Sal rats is driven by a loss of GABA-GABA connectivity in vSub. For example, when the MS is activated in Sal or intact rats, GABA from the MS GABAergic projection to vSub^15,38^ activates GABA_A_ receptors on interneurons, which tend to be more sensitive to GABA than primary neurons.^39–41^ This would inhibit vSub interneurons, disinhibiting pyramidal cells, and combined with MS cholinergic projections activating pyramidal cells,^14,15,42^ would result in an increase in hippocampal activity. This is consistent with our previous finding that TTX inactivation of the vSub prevented MS-mediated changes in both VTA and SNc.^13^ When the MS is activated in MAM rats, GABA release in vSub would likely reach both interneurons and pyramidal cells because of the loss of higher-sensitivity interneuron targets. This would lead to an opposite decrease in pyramidal cell activity, possibly explaining how DA activity, and subsequently AIH, could be opposite in MAM compared to Sal animals. Furthermore, bicuculline infusion into the vSub, which would prevent GABA inhibition of both interneurons and pyramidal cells, brings DA activity in SNc and AIH back to baseline levels (see Figs. 4 and 5).

### Proposed mechanism for early epoch effects on AIH

NMDA activation of the MS reduced baseline open field locomotor behavior in both Sal and MAM rats (Fig. 3, minute 5), suggesting it was unaffected by MAM treatment. Additionally, this effect was not blocked by bicuculline infusion into the vSub (Fig. 4) suggesting that it is not likely regulated via the previously described vSub to SNc pathway (Supplementary Figure 2). Therefore, since MAM treatment is known to increase DA activity in the VTA^29,36^ and vSub bicuculline infusions are known to affect MS-driven DA activity changes in the SNc,^13^ it is possible that this baseline locomotor effect is not driven by changes within the DA system. The reduction in locomotor behavior following MS activation is, however, in line with previous studies suggesting that combined lesion of the septohippocampal GABAergic and cholinergic projections results in an increase in baseline locomotor behavior.^43^ One possibility is that locomotion was decreased due to an increase in grooming behavior. This notion is supported by the finding that MS activation resulted in a greater degree of amphetamine-induced stereotypy that was similarly unaffected by MAM treatment (Supplementary Figure 1).

### Behavioral implications

It has been hypothesized that the primary behavioral consequence of MS activation, which leads to an increase in VTA DA activity and a decrease in SNc DA activity in intact animals,^13^ could be an attenuation of SNc-driven impulsive responding^34,44,45^ in favor of VTA-driven goal directed responding.^34,45^ This could produce a “stop and consider alternate possibilities” mode of responding in situations where reward contingencies had changed. This concept is supported by data showing that septohippocampal lesions increase perseverative responding and proactive interference and decrease fear extinction.^19,46^ The results in this paper indicate that MAM-treated animals display the opposite pattern of midbrain DA activity and a significant increase in AIH in response to MS activation. This could suggest that the behavioral effect of MS activation in MAM rats would be to act impulsively, choosing without consideration for changing situational and environmental contingencies. Interestingly, this behavioral disposition is a hallmark of MAM rats^47^ and schizophrenia patients^48^ as demonstrated clearly in reversal learning and cognitive flexibility tasks. Therefore, it is possible, although speculative, that disruptions in the MS’s regulation of the DA system could play a role in the cognitive flexibility deficits seen in schizophrenia patients.

### Selective MS activation as a potential treatment strategy

An interesting finding from this paper is that MS activation reduced VTA DA activity in MAM rats back to intact baseline levels, ~1.0 active DA neuron per recording track. This probably occurred due to an increase in pyramidal cell inhibition (see proposed circuit section above), thus compensating for the interneuron loss that is a hallmark in MAM rats.^28,37^ This might suggest that manipulation of the MS-vSub pathway could have the potential to reverse the hyperactive DA state seen in MAM^3,29^ and schizophrenia.^49–51^ However, the decrease in VTA DA population activity was coincident with a potentially pathological increase in SNc DA activity and AIH. Such a condition has the potential to result in an increase in habit-related, impulsive responding that is typical of dorsal striatal DA release (see behavioral implications above).^34,44,45^ Therefore, it is unlikely that general activation of the MS would be cognitively beneficial in MAM animals or patients. Alternatively, it was previously shown that MS-mediated VTA and SNc DA activity could be selectively modulated via different sub-regions of the ventral pallidum and different neurotransmitter inputs to vSub.^13^ Furthermore, we demonstrated that infusion of bicuculline into the vSub, which prevented the MS-mediated increase in SNc DA activity without affecting the VTA,^13^ was able to reverse the behavioral deficit in AIH, bringing MAM/NMDA rats back to Sal/dPBS locomotion levels. Therefore, selective manipulation of the MS to midbrain pathway might be able to reverse the hyperactive DA state in the VTA without increasing DA activity in the SNc, potentially alleviating the hyperdopaminergic state seen in the disease without inducing other non-specific effects.

### Summary

This manuscript demonstrates that the MS-mediated regulation of DA neuron activity in the VTA and SNc and AIH is disrupted in MAM-treated rats, leading to an opposite response in both DA activity and behavior compared to Sal and intact rats. The elevation in SNc DA activity and AIH were both reversed by blocking GABAergic inputs in the vSub, implicating the ventral hippocampal GABAergic system as a primary locus for disruption in the model. Based on the known role of the MS in cognition, these data suggest that a disruption in the MS’s regulation of DA activity could play a role in the expression of hallmark schizophrenia deficits, such as increased impulsivity and decreased cognitive flexibility; thus highlighting selective manipulation of the MS as a potential locus for therapeutic efficacy in schizophrenia.

## Methods

### Animals

Timed pregnant Sprague Dawley rats were obtained from Envigo and treated with MAM (20 mg/kg i.p.) or Sal at gestational day 17. Male MAM or Sal pups were weaned and pair-housed with ad libitum access to food and water in a temperature and humidity controlled room until used for experiments at adulthood (PD60–90). Experimental procedures were approved by the Institutional Animal Care and Use Committee of the University of Pittsburgh according to National Institute of Health Guide for the Care and Use of Laboratory Animals.

### Electrophysiological recordings

MAM and Sal rats were anesthetized with an initial dose of chloral hydrate (400 mg/kg i.p.), secured to a stereotaxic frame (Kopf), and implanted with a guide cannula in the (MS; AP: +0.5, ML: 0, DV: −5.8 mm from bregma) as previously described.^13^ NMDA (0.75 µg in 0.2 µL; Sigma Aldrich) mixed in Dulbecco’s phosphate buffered saline (dPBS; Sigma Aldrich) or dPBS alone was then infused (0.5 µL/min) just prior to electrophysiological recording of the VTA or SNc. Chemical stimulation was used for these experiments to produce stable, long-duration excitation, and the dose of NMDA used corresponded with previous studies.^13,21,22,30,52^ For experiments involving intrasubiculum infusions, bicuculline (12.5 ng in 0.5 µL) was infused (AP: −6.0, ML: 4.5 in mm from bregma, DV: −8.5 in mm from skull) just prior to NMDA activation of the MS in separate animals, using the same procedure.

Following drug infusions, glass recording electrodes were lowered into the VTA or SNc in nine sequential vertical tracks (see Fig. 1). The number of active DA neurons recorded within an animal was averaged across the total number of tracks recorded (neurons/track) to yield a measure of population activity. Coordinates were determined using an atlas.^53^ The recording pattern has been previously described^13,54^ and uses well-established identification criteria to identify DA neurons.^55,56^ Once identified, neurons were recorded for 3 minutes and assessed for firing rate and burst firing properties (burst = action potentials occurring with an interspike interval of ≤80 ms and terminating with an interspike interval of >160 ms).^57^ At the conclusion of the ninth track, electrophoretic ejection of Chicago sky blue dye marked the recording location for histological confirmation of electrode site.

### AIH and stereotypy

MAM and Sal rats were implanted with guide cannulae into the MS or into both the MS and vSub as previously described.^13^ Following a one week recovery period, rats were infused with either NMDA or dPBS in the MS (Fig. 3) or NMDA or dPBS in the MS and bicuculline in the vSub (Fig. 4) 10 min before being placed in an open-field arena (Coulborn). Locomotor activity was measured by beam breaks in the *x–y* plane and analyzed in 5 min epochs. Systemic injections of d-amphetamine (0.75 mg/kg) were given after a 30 min baseline period, and the post-amphetamine session lasted for 60 min. This allowed us to determine the effect of long-duration MS activation on both baseline locomotion and the transient hyperlocomotion induced by amphetamine injection. Following the locomotor test, rats were given an additional injection of amphetamine (2 mg/kg), placed in a small metal cage, and observed to measure their stereotyped behavior. Stereotypic behaviors; including oral stereotypy, sniffing, rearing, vigorous grooming, and climbing, were scored three times during each 5-min epoch on a 0–3 scale.^58,59^ This yielded a value between 0 and 9 for each 5 min bin during the 45-min observation period, with 9 indicating the highest degree of stereotyped behavior.

### Histology

Following each experiment, MAM and Sal rats were sacrificed for histological verification of cannula and electrode placement according to a stereotaxic atlas^53^ as previously described.^13^

### Statistical analysis

Detection and analysis of DA neuron activity was performed using LabChart and NeuroExplorer, and locomotor behavior was recorded using TruScan software. Dependent measures included DA population activity, neuron firing rate, percentage of spikes occurring in bursts, and total distance traveled (cm). All measures (reported as mean ± SEM), were analyzed by ANOVA. For electrophysiology experiments a one-way ANOVA was performed using intracranial drug condition (combination of intracranial drug infusions, example MS NMDA or MS NMDA/vSub bicuc) as a between-subjects variable. AIH was analyzed using a two-way ANOVA with intracranial drug condition (combination of intracranial NMDA/dPBS or bicuculline infusions) as a between-subjects variable and time point (5 min epochs) as a within-subjects variable. Post-hoc analyses were performed using the Tukey’s test. Significance is defined as *P* < 0.05 (IBM SPSS Statistics 22).

## Electronic supplementary material

Supplemental Figures

## Data Availability

The datasets generated during and/or analyzed during the current study are available from the corresponding author (D. Bortz, dmb164@pitt.edu) on reasonable request.

## Electronic supplementary material

**Supplementary information** accompanies the paper on the *npj Schizophrenia* website (10.1038/s41537-018-0059-3).
