# Supplementary material for: Medial septum activation produces opposite effects on dopamine neuron activity in the ventral tegmental area and substantia nigra in MAM vs. normal rats
Source: NPJ Schizophr. 2018 Sep 3;4:17. doi: 10.1038/s41537-018-0059-3 (PMC6120917; doi:10.1038/s41537-018-0059-3)
Supplement: Supplementary file 1 — Supplemental Figures [file 41537_2018_59_MOESM1_ESM.pdf]

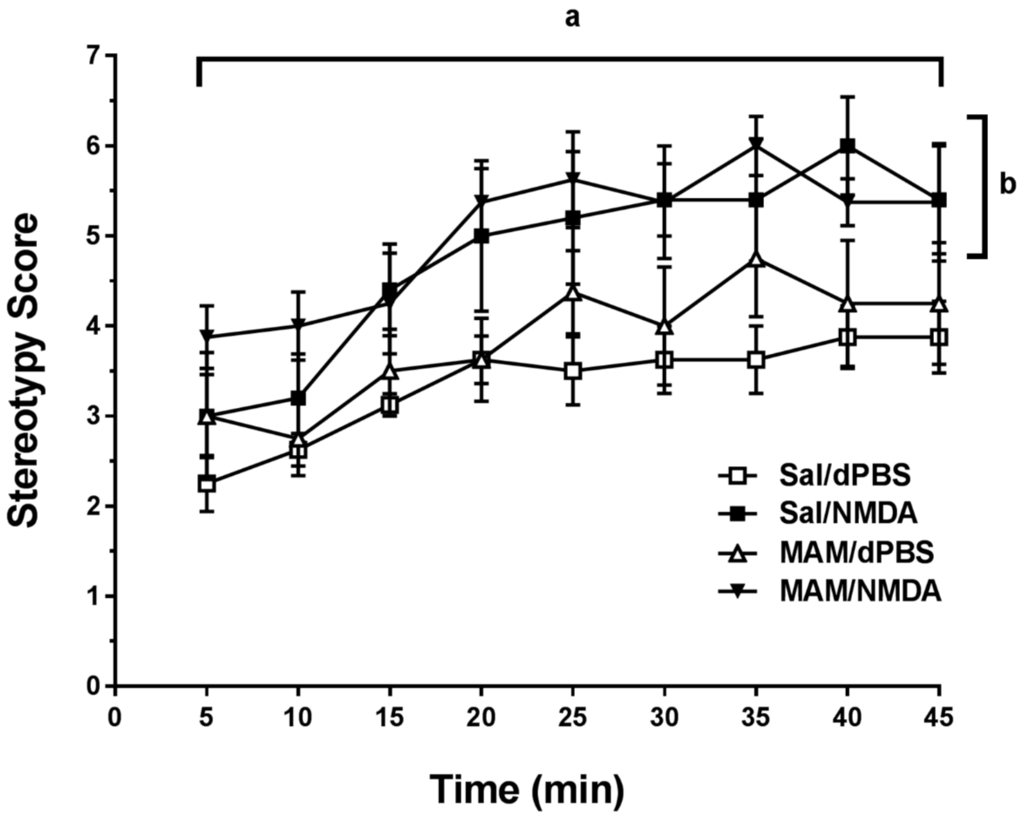

Supplementary Figure 1: MS activation does not increase stereotyped behavior in MAM versus Sal-treated rats. Amphetamine injection (2 mg/kg) following MS activation (NMDA, 0.75 µg in 0.2 µL or dPBS) results in a significant increase in stereotypy over time (a= $F_{8, 200} = 16.74$, $P < 0.0001$) that is greater in NMDA-treated versus dPBS-treated rats (b= $F_{3, 25} = 4.967$, $P = 0.0077$). However, this does not interact with MAM treatment ($F_{24, 200} = 0.6961$, $P = 0.85$), as MAM/NMDA rats do not differ from Sal/NMDA rats at any time point (all P's > 0.05).

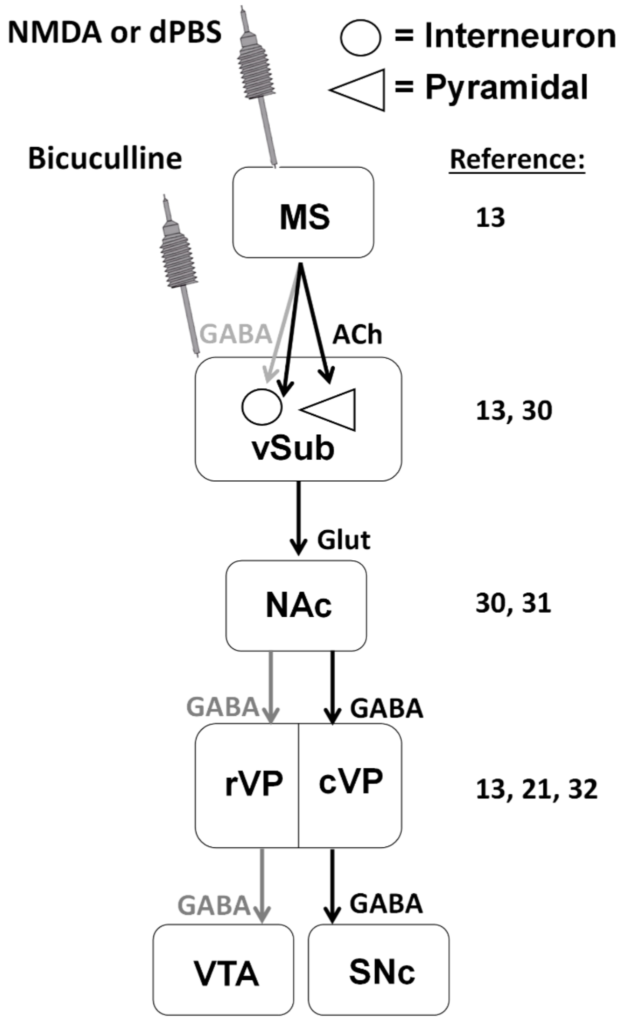

Supplementary Figure 2: Proposed multi-synaptic pathway from MS to midbrain and supporting references. The medial septum (MS) contacts the ventral subiculum (vSub)[38] through projections that are predominately cholinergic and GABAergic.[15] GABAergic projections largely target GABAergic interneurons[25], whereas the cholinergic projections target both pyramidal neurons and interneurons[42], leading to activation of the vSub.[13] The vSub activates the nucleus accumbens (NAc)[30,34], which inhibits the ventral pallidum (VP).[21,32] VP regulation of midbrain DA neuron activity is functionally split along the rostral/caudal axis (rVP, cVP), with the rVP selectively modulating population activity changes in the VTA and the cVP selectively modulating changes in the SNc.[13] Sites of pharmacological manipulation and what was infused are indicated.
